# Maternal Supplementation With Different Probiotic Mixture From Late Pregnancy to Day 21 Postpartum: Consequences for Litter Size, Plasma and Colostrum Parameters, and Fecal Microbiota and Metabolites in Sows

**DOI:** 10.3389/fvets.2022.726276

**Published:** 2022-02-08

**Authors:** Li Han, Md. Abul Kalam Azad, Pan Huang, Wei Wang, Wenming Zhang, Francois Blachier, Xiangfeng Kong

**Affiliations:** ^1^Hunan Provincial Key Laboratory of Animal Nutritional Physiology and Metabolic Process, National Engineering Laboratory for Pollution Control and Waste Utilization in Livestock and Poultry Production, Institute of Subtropical Agriculture, Chinese Academy of Sciences, Changsha, China; ^2^The Institute of Cell Transplantion and Gene Therapy, Centra-South University, the Engineering Center for Xenotransplantation, Changsha, China; ^3^Evonik (China) Co., Ltd., Beijing, China; ^4^UMR PNCA, INRAE, AgroParisTech, Université Paris-Saclay, Paris, France

**Keywords:** fecal microbiota, litter size, metabolites, pregnant sows, probiotics

## Abstract

The present study determined the effects of different probiotic mixture supplementation to sows from late pregnancy to day 21 postpartum on reproductive performance, colostrum composition, plasma biochemical parameters, and fecal microbiota and metabolites. A total of 80 pregnant sows were randomly assigned to one of four groups (20 sows per group). The sows in the control group (CON group) were fed a basal diet, and those in the BS-A+B, BS-A+BL, and BS-B+BL groups were fed basal diets supplemented with 250 g/t of different probiotic mixture containing either 125 g/t of *Bacillus subtilis* A (BS-A), *Bacillus subtilis* B (BS-B), and/or *Bacillus licheniformis* (BL), respectively. The trial period was from day 85 of pregnancy to day 21 postpartum. The results showed that different dietary probiotic mixture supplementation increased (*P* < 0.05) the average weaning weight and average daily gain of piglets, while dietary BS-A+BL supplementation increased the number of weaned piglets (*P* < 0.05), litter weight (*P* = 0.06), litter weight gain (*P* = 0.06), and litter daily gain (*P* = 0.06) at weaning compared with the CON group. Different dietary probiotic mixture supplementation improved (*P* < 0.05) the colostrum quality by increasing the fat and dry matter concentrations, as well as the protein and urea nitrogen concentrations in the BS-A+BL group. Dietary probiotic mixture BS-B+BL increased the plasma total protein on days 1 and 21 postpartum while decreased the plasma albumin on day 1 postpartum (*P* < 0.05). In addition, the plasma high-density lipoprotein-cholesterol was increased in the BS-A+B and BS-B+BL groups on day 21 postpartum, while plasma ammonia was decreased in the BS-A+B and BS-A+BL groups on day 1 and in the three probiotic mixtures groups on day 21 postpartum (*P* < 0.05). Dietary supplementation with different probiotic mixture also modified the fecal microbiota composition and metabolic activity in sows during pregnancy and postpartum stages. Collectively, these findings suggest that maternal supplementation with *Bacillus subtilis* in combination with *Bacillus licheniformis* are promising strategies for improving the reproductive performance and the overall health indicators in sows, as well as the growth of their offspring.

## Introduction

Reproductive performance can be influenced by the health status of sows during pregnancy, and such a parameter is closely associated with the economic efficiency of pig farms (1). However, sows are susceptible to various stress factors (including factors associated with service staff, environment, physiological stages, etc.) during pregnancy and lactation. Such situation of stress may cause imbalance of intestinal microbiota composition and metabolic activity, lower nutrient utilization, and lead to sows body weight loss (2). The gut microbiota composition of sows during pregnancy and lactation can impact the enteric nutrient absorption and immunity (3), which consequently influences the body weight (BW) of piglets at birth and weaning, the number of piglets born alive, and the number of living piglets at weaning (4). Moreover, the BW loss of sows during lactation can influence the lactation performance, as well as the subsequent weaning-to-service interval and reproductive cycle (5). Therefore, in order to maximize the reproductive potential and the body health of sows, such objectives might be achieved by different dietary strategies, including supplementation with antibiotics, probiotics, prebiotics, and enzymes in sow diets (6). Recent concerns regarding antibiotic resistance in animals and humans has led to the use of antibiotic alternatives such as probiotic strains in livestock production. Such alternative has attracted increased attention to improve the reproductive performance and overall health of animals.

The most commonly used probiotics in livestock production include the *Bifidobacterium, Lactobacillus, Bacillus* spp., *Enterococcus* spp., and *Saccharomyces cerevisiae* (7). Among these probiotics, *Bacillus* spp. is differentiated by its ability to survive in the intestinal tract, form spores, secrete bacteriostatic substances, withstand adverse conditions of feed processing, and maintain stability. Moreover, *Bacillus* spp. produces different kinds of digestive enzymes and stimulates peristalsis of the host intestine, thereby enhancing nutrient digestion (8, 9). Therefore, it is deemed to be a beneficial feed additive for animal intestinal health (10). *Bacillus* spp. are also widely used as probiotics in humans, as they may bring a health benefit to the host gastrointestinal physiology (11). Concurrently, Cai et al. (12) have also shown that dietary *Bacillus* spp. supplementation has positive effects on pigs, such as improving growth performance and feed conversion ratio, reducing the incidence of diarrhea and mortality, as well as increasing the BW and number of piglets born alive and kept alive up to weaning time. Moreover, previous studies also revealed that a probiotic mixture of *Bacillus subtilis* and *Bacillus licheniformis* in growing-finishing pigs increased the digestibility and fecal *Lactobacillus* counts while decreased fecal NH_3_ and total mercaptan emissions (13). The decreased NH_3_ concentration is also considered beneficial for colonocyte mitochondrial energy metabolism, as this bacterial metabolite inhibits oxygen consumption in colonocytes when present in excess (14).

Although various *Bacillus* spp. are used as probiotics for animals and humans, the mixture of different *Bacillus* strains (such as *B. subtilis* and *B. licheniformis*) has been little studied in pigs, and the mechanisms involved in the effects observed are not yet fully understood. In addition, most studies and applications of *Bacillus* spp. are mostly concentrated on the stages of piglets at nursery, weaned piglets, and growing pigs. However, the studies are relatively limited on the effects of *Bacillus* spp. used during pregnancy and lactation, regarding the impact on the sow reproductive performance and the profiles of the sow's intestinal microbiota. In addition, the effects of maternal supplements on their offspring piglets have been little documented. Our previous study found that dietary supplementation with a probiotic mixture of *B. subtilis* and *B. licheniformis* to piglets at weaning could improve several indicators of intestinal health through improving intestinal morphology and altering intestinal microbiota and metabolite concentrations (15). In addition, dietary supplementation with *B. subtilis* increased the amounts of intestinal microbes with presumed beneficial effects, and the fecal concentrations of several bioamines and short-chain fatty acids (SCFA) of perinatal sows (15, 16). In this context, we hypothesized that dietary probiotic mixture (*B. subtilis* A, BS-A; *B. subtilis* B, BS-B; and/or *B. licheniformis*) supplementation from late pregnancy to day 21 postpartum would be beneficial for sow health and thus influence their reproductive performance. The BS-A is a product containing a single *B. subtilis* strain and has a strong capability of *Clostridium perfingens* inhibition. The BS-B is a product containing a single pure *Bacillus* strain with strong *Escherichia coli F18* inhibition ability. Both strains show significant pathogens inhibition through multiple secondary metabolites production. Moreover, *B. licheniformis* is a product containing a single pure *B. licheniformis* strain, which has the potential to improve intestinal morphology in broilers. Therefore, the combination of these strains might have synergic beneficial effects in animals. Thus, the present study was conducted to determine the effect of dietary supplementation with probiotic mixture containing *B. subtilis* and/or *B. licheniformis* from late pregnancy to day 21 postpartum on reproductive performance, biochemical parameters in blood and colostrum, and intestinal microbiota composition and their metabolites.

## Materials and Methods

### Animals, Housing, and Treatments

A total of 80 Large White sows close to day 85 of pregnancy with 2–4 parities were used and randomly assigned to one of four groups (20 sows per group). The sows in the control group were fed the basal diet (CON group), and those in the experimental groups received the basal diet supplemented with 250 g/t complex probiotics (4.0 × 10^9^ CFU/kg). The diets of the BS-A+B, BS-A+BL, and BS-B+BL groups contained 125 g/t *B. subtilis* A (BS-A) + 125 g/t *B. subtilis* B (BS-B), 125 g/t BS-A + 125 g/t *B. licheniformis* (BL), and 125 g/t BS-B + 125 g/t BL, respectively. The complex probiotics were prepared by *Evonik* (*China*) *Co., Ltd*. The trial lasted from day 85 of pregnancy to day 21 postpartum.

The pregnant sows were housed in individual pens (2.50 × 0.85 m) during late pregnancy (days 85–110), and were moved to the farrowing facilities (2.50 × 2.70 m) on day 110 of pregnancy, where they were housed individually with a hard plastic slatted bedding, together with their litters until weaning. The room temperature was maintained at 21–24°C with 60% relative humidity. In addition, heating lights were used to maintain the temperature of the piglets. The sows were fed a pregnancy diet between days 85 to 107 of pregnancy and a lactation diet from day 108 of pregnancy to day 21 postpartum. The sows were fed twice daily (8:00 a.m. and 5:00 p.m.) with ~2.0–3.0 kg of diets and changed according to their body condition. Sows and piglets had available *ad libitum* access to water throughout the trial *via* the individual nipple. The composition and nutrient levels of the basal diets for pregnant and lactating sows are shown in Table 1.

**Table 1:** Ingredients and nutrient levels of basal diets of sows during late pregnancy and lactation (as-fed basis).

**Items**	**Pregnancy diet**	**Lactation diet**
**Ingredients (%)**		
Corn	60.30	58.65
Wheat bran	23.50	5.00
Wheat flour		2.00
Soybean oil		4.00
Soybean meal	12.00	20.50
Enzymic protein powder		3.00
Fish meal		2.50
L-Lysine·HCl	0.12	0.15
L-Threonine	0.03	0.05
L-Valine		0.10
Anti-mildew agent	0.05	0.05
Pregnancy compound premix^a^	4.00	
Lactation compound Premix^b^		4.00
Total	100.00	100.00
Nutrient levels (%)^c^		
Digestible energy (MJ/kg)	15.23	15.56
Dry matter	98.00	97.74
Crude fiber	3.60	3.54
Crude protein	14.17	19.78
Lysine	0.98	1.53
Methionine	0.12	0.16
Threonine	0.68	0.99

a *Provided the following for one kilogram diet: VA 10,000 IU, VD 2,500 IU, VE 100 IU, VK 2.0 mg, VB_2_ 10 mg, VB_6_ 1.0 mg, VB_12_ 50 μg, choline chloride 1,500 mg, Fe 80 mg, Cu 20 mg, Zn 100 mg, Mn 45 mg, I 0.7 mg, and Se 0.25 mg*.

b *Provided the following for one kilogram diet: VA 15,000 IU, VD 3,200 IU, VE 50 IU, VK 4.0 mg, VB_1_ 4.0 mg, VB_2_ 10 mg, VB_6_ 3.0 mg, VB_12_ 20 μg, choline chloride 800 mg, Fe 120 mg, Cu 20 mg, Zn 112 mg, Mn 24 mg, I 0.5 mg, and Se 0.4 mg*.

c *Digestible energy is a calculated value, and others are analyzed values*.

### Sample Collection and Preparation

On day 1 postpartum, the litter size, number of born alive, and litter weight were recorded, as well as the number and weight of weaned piglets per litter on day 21 postpartum, to calculate the daily gain of litters at weaning and average daily gain of piglets. The backfat thickness was measured at the level of the last rib at 5–8 cm from the midline of each sow using ultrasonography (Renco Lean-Meater®, Minneapolis, MN, USA) on days 85 and 112 of pregnancy, and again on day 21 postpartum. The colostrum samples (~10 mL) of eight sows per group were collected within 12 h after farrowing and stored at −80°C for the analysis of colostrum composition. The fresh fecal samples were randomly collected in 50 mL sterile centrifuge tubes from eight sows per group on days 100 and 112 of pregnancy and on days 7, 14, and 21 postpartum, and then stored at −20°C for analysis of the microbiota composition and metabolite concentrations. On days 1 and 21 postpartum, the blood samples were randomly collected from the precaval vein into 10 mL heparin coated-tubes, and plasma was separated by centrifuging at 3,500 × g for 10 min at 4°C and immediately stored at −20°C for the analysis of biochemical parameters.

### Analysis of Plasma Biochemical Parameters

The plasma biochemical parameters, including total protein (TP), albumin (ALB), urea nitrogen (UN), ammonia (AMM), alkaline phosphatase (ALP), triglyceride (TG), total cholesterol (TC), high-density lipoprotein-cholesterol (HDL-C), and low-density lipoprotein-cholesterol (LDL-C) were measured using a Roche Automatic Biochemical Analyzer (Cobas c311, F. Hoffmann-La Roche Ltd, Basel, Switzerland) and commercially available kits (F. Hoffmann-La Roche Ltd, Basel, Switzerland).

### Analysis of Colostrum Composition

The colostrum composition, including somatic cells, milk fat, milk protein, lactose, urea nitrogen, defatted dry matter, and total dry matter, were determined using MilkoScan FT+200 Type 76150 (FOSS electric, Hilleroed, Denmark).

### DNA Extraction and Analysis of Fecal Microbiota Quantity

Total microbial DNA was extracted and purified according to the manufacturer's instructions of QIAamp DNA Stool Mini Kit (QIAgen, Hilden, Germany). The concentration of each extracted DNA was measured using a NanoDrop ND-1000 instrument (NanoDrop Technologies Inc., Wilmington, DE, USA) and stored at −80°C. An absorption ratio (260/280 nm) of all samples within 1.8–2.0 was deemed to be of sufficient purity to be used for subsequent analyses. The 16S rRNA gene sequences of *Bifidobacterium* spp., *Clostridium* cluster IV, *Escherichia coli, Firmicutes, Lactobacillus*, and total bacteria were cloned into the pMD19-T vector (17). Gene sequences by references (18) were amplified from total DNA using the primers listed in Table 2. A total of six clones with 16S rRNA gene sequences belonging to different taxa were used as templates to test primer specificity. Standard curves were constructed with DNA from representative species of a concentration range of 10^2^-10^10^ DNA copies per mL using 384-well plates in the Lightcycler® 480 instrument II (Applied Biosystems, Carlsbad, CA, USA). The microbial DNA extracted from the fecal samples and specific DNA from recombinant microbiota were quantified by RT-PCR. Reaction conditions were at 50°C for 2 min, an initial denaturation step at 95°C for 5 min, and then 20 s denaturation at 94°C for 40 cycles, primer annealing at a species-specific temperature for 30 s, and primer extension at 60°C for 1 min (19). The specific primers for RT-PCR were synthesized by Sangon Biotech (Shanghai) Co., Ltd. Data were analyzed using the Roche Lightcycler 480 software 1.5.0. Microbiota quantities were expressed as a logarithm of the number of microbe copies contained per gram of samples [lg (copies/g)].

**Table 2:** Group-specific primer sequences for bacteria.

**Bacteria**	**Sequence (5^**′**^-3^**′**^)**	**Product size (bp)**
*Bifidobacterium*	F: TCGCGTCYGGTGTGAAAG	128
	R: GGTGTTCTTCCCGATATCTACA	
*Clostridium cluster* IV	F: GCACAAGCAGTGGAGT	240
	R: CTTCCTCCGTTTTGTCAA	
*Escherichia coli*	F: CATGCCGCGTGTATGAAGAA	95
	R: CGGGTAACGTCAATGAGCAAA	
Firmicutes	F: GGAGYATGTGGTTTAATTCGAAGCA	126
	R: AGCTGACGACAACCATGCAC	
*Lactobacillus*	F: AGCAGTAGGGAATCTTCCA	345
	R: ATTCCACCGCTACACATG	
Total bacteria	F: GTGSTGCAYGGYYGTCGTCA	123
	R: ACGTCRTCCMCNCCTTCCTC	

### Analysis of Fecal Bacterial Metabolites

The concentrations of fecal SCFA, including acetate, propionate, butyrate, isobutyrate, valerate, and isovalerate, were measured as described previously by Zhou et al. (20). The fresh fecal samples (0.900–1.000 g) were homogenized and centrifuged in sealed tubes at 10,000 × g for 10 min at 4°C. A mixture of the supernatant fluid and 25% metaphosphoric acid solution (1 mL: 0.25 mL) were then filtered through a 0.45-μm polysulfone microporous membrane filter and analyzed using Agilent 6890 gas chromatography (Agilent Technologies, Inc, Palo Alto, CA, USA) (21). The concentrations of bioamines, including tryptamine, putrescine, cadaverine, 1,7-heptyl diamine, tyramine, spermidine, and spermine, were measured as described previously by Kong et al. (22).

### Statistical Analyses

Statistical data analysis was performed with one-way ANOVA using SPSS 18.0 software (SPSS, Inc, Chicago, IL, USA). Levene's test for homogeneity of variance was used, followed by Duncan's multiple-range test (in the case of variance homogeneity). Values are expressed as means ± standard error (SE). *P*-values < 0.05 were taken to indicate statistical significance, with a trend toward significance at 0.05 ≤ *P* < 0.10.

## Results

### Reproductive Performance

The effects of different dietary probiotic mixture supplementation on the reproductive performance of sows are presented in Table 3. The average weaning weight and average daily gain of weaned piglets were increased (*P* < 0.05) by supplementing the sows' diet with different probiotic mixture. However, dietary supplementation with different probiotic mixture did not affect neither the litter size nor the number of piglets born alive. Also, the litter weight at birth remains unchanged compared with the CON group. The number of weaned piglets was higher (*P* < 0.05) in the BS-A+BL group compared with the CON and BS-A+B groups. In addition, the BS-A+B group displayed a trend for an increased (*P* = 0.06) average piglets' birth weight. Moreover, the BS-A+BL group also displayed a trend for an increased litter weight (*P* = 0.07), litter weight gain (*P* = 0.06), and litter daily gain (*P* = 0.06) at weaning.

**Table 3:** Effects of dietary probiotic mixture supplementation on reproductive performance of sows.

**Items**	**CON group**	**BS-A+B group**	**BS-A+BL group**	**BS-B+BL group**	***P-*values**
Litter size (*n*)	11.84 ± 0.68	10.32 ± 0.54	12.42 ± 0.86	11.42 ± 0.66	0.19
Born alive (*n*)	11.53 ± 0.73	10.26 ± 0.52	12.05 ± 0.79	11.26 ± 0.63	0.30
Weaned piglets (*n*)	10.00 ± 0.26^b^	9.85 ± 0.25^b^	10.90 ± 0.28^a^	10.40 ± 0.26^ab^	0.02
Litter weight at birth (kg)	17.07 ± 0.95	17.25 ± 0.74	18.56 ± 1.03	17.99 ± 0.72	0.60
Average birth weight (kg)	1.50 ± 0.04	1.70 ± 0.05	1.57 ± 0.05	1.64 ± 0.06	0.06
Litter weight at weaning (kg)	61.72 ± 3.02	68.24 ± 2.69	72.02 ± 2.47	69.10 ± 2.52	0.06
Litter weight gain at weaning (kg)	46.65 ± 2.47	52.66 ± 2.36	55.20 ± 2.07	52.20 ± 2.11	0.06
Litter daily gain at weaning (kg/d)	2.22 ± 0.12	2.51 ± 0.11	2.63 ± 0.10	2.49 ± 0.10	0.06
Average weaning weight (kg)	6.15 ± 0.16^b^	7.09 ± 0.17^a^	6.75 ± 0.18^a^	6.72 ± 0.16^a^	<0.01
Average daily gain (kg)	0.22 ± 0.01^b^	0.26 ± 0.01^a^	0.25 ± 0.01^a^	0.24 ± 0.01^a^	<0.01

*Data are presented as means with SE (n = 20)*.

a, b *Mean values in the same row with different superscripts were significantly different (P < 0.05). The BS-A+B, BS-A+BL, and BS-B+BL groups contained 125 g/t Bacillus subtilis A (BS-A), 125 g/t Bacillus subtilis B (BS-B), and/or 125 g/t Bacillus licheniformis (BL), respectively*.

### Backfat Thickness and Colostrum Composition

The effects of different dietary probiotic mixture supplementation on the backfat thickness of sows are presented in Table 4. The backfat thickness of the BS-A+BL group was increased (*P* < 0.05) from day 85 to day 112 of pregnancy compared with the CON and BS-B+BL groups. However, there were no significant changes in the backfat thickness of sows from day 112 of pregnancy to day 21 postpartum among the different dietary treatment groups.

**Table 4:** Effects of dietary probiotic mixture supplementation on backfat thickness of sows.

**Items**	**CON group**	**BS-A+B group**	**BS-A+BL group**	**BS-B+BL group**	***P-*values**
**Backfat thickness (mm)**					
Day 85 of pregnancy	18.75 ± 1.02	19.85 ± 1.15	18.50 ± 0.88	19.30 ± 0.95	0.79
Day 112 of pregnancy	19.55 ± 0.87	21.25 ± 1.07	21.45 ± 0.94	21.00 ± 1.01	0.51
Day 21 postpartum	16.55 ± 0.80	17.35 ± 0.96	19.20 ± 0.82	18.00 ± 0.90	0.19
**Backfat thickness changes (mm)**					
Day 85 to day 112 of pregnancy	1.71 ± 0.22^b^	2.64 ± 0.46^ab^	3.39 ± 0.39^a^	2.00 ± 0.40^b^	0.01
Day 112 of pregnancy to day 21 postpartum	−3.33 ± 0.61	−4.39 ± 0.50	−3.31 ± 0.50	−3.76 ± 0.58	0.47

*Data are presented as means with SE (n = 20)*.

a, b *Mean values in the same row with different superscripts were significantly different (P < 0.05). The BS-A+B, BS-A+BL, and BS-B+BL groups contained 125 g/t Bacillus subtilis A (BS-A), 125 g/t Bacillus subtilis B (BS-B), and/or 125 g/t Bacillus licheniformis (BL), respectively*.

The effects of different dietary probiotic mixture supplementation on nutrient compositions of colostrum are summarized in Table 5. Compared with the CON group, the concentrations of milk fat and total dry matter in colostrum were increased (*P* < 0.05) when sows were supplemented with different probiotic mixture. The concentrations of protein and UN of colostrum were higher (*P* < 0.05) in the BS-A+BL group compared with the CON group.

**Table 5:** Effects of dietary probiotic mixture supplementation on nutrient composition of colostrum in sows.

**Items**	**CON group**	**BS-A+B group**	**BS-A+BL group**	**BS-B+BL group**	***P*-values**
Somatic cells (×10^3^ piece/mL)	1,399 ± 373.8	1,591 ± 369.1	1,324 ± 472.5	2,337 ± 1,134.3	0.70
Fat (%)	2.66 ± 0.23^b^	3.89 ± 0.38^a^	3.92 ± 0.21^a^	3.66 ± 0.46^a^	0.04
Protein (%)	14.76 ± 0.42^b^	15.59 ± 0.64^ab^	17.19 ± 0.37^a^	16.23 ± 0.68^ab^	0.03
Lactose (%)	3.96 ± 0.13	3.86 ± 0.18	3.72 ± 0.07	3.62 ± 0.12	0.30
Urea nitrogen (mg/dL)	50.13 ± 2.08^b^	56.13 ± 2.41^ab^	62.25 ± 2.91^a^	56.75 ± 2.3^ab^	0.02
Defatted dry matter (%)	22.73 ± 0.34	23.48 ± 0.5	24.41 ± 0.46	23.87 ± 0.57	0.11
Total dry matter (%)	28.62 ± 0.51^b^	30.76 ± 0.69^a^	31.65 ± 0.41^a^	30.91 ± 0.92^a^	0.02

*Data are presented as means with SE (n = 8)*.

a, b *Mean values in the same row with different superscripts were significantly different (P < 0.05). The BS-A+B, BS-A+BL, and BS-B+BL groups contained 125 g/t Bacillus subtilis A (BS-A), 125 g/t Bacillus subtilis B (BS-B), and/or 125 g/t Bacillus licheniformis (BL), respectively*.

### Plasma Biochemical Parameters

The effects of different dietary probiotic mixture supplementation on plasma biochemical parameters of sows are presented in Table 6. On day 1 postpartum, the plasma ALB concentration was decreased (*P* < 0.05) in the BS-B+BL group compared with the other three groups, while the plasma TP concentration was increased (*P* < 0.05) in the BS-B+BL group compared with the CON group. In addition, the plasma ALP activity was decreased (*P* < 0.05) in the BS-A+B group compared with the CON and BS-B+BL groups, and the plasma AMM concentration was decreased (*P* < 0.05) in the BS-A+B and BS-A+BL groups compared with the other two groups on day 1 postpartum. Moreover, the plasma UN concentration was higher (*P* = 0.06) in the BS-A+BL group on day 1 postpartum. On day 21 postpartum, the plasma AMM concentration was decreased (*P* < 0.05) in the three probiotic mixture groups compared with the CON group, while the plasma HDL-C concentration was increased (*P* < 0.05) in the BS-A+B and BS-B+BL groups compared with the other two groups. Moreover, the plasma TC concentration (*P* = 0.06) in the BS-A+B group and TP concentration (*P* = 0.06) in the BS-B+BL group tended to increase on day 21 postpartum.

**Table 6:** Effects of dietary probiotic mixture supplementation on plasma biochemical parameters of sows.

**Items**	**Day postpartum**	**CON group**	**BS-A+B group**	**BS-A+BL group**	**BS-B+BL group**	***P*-values**
ALB (g/L)	1	41.76 ± 0.63^a^	42.39 ± 0.83^a^	43.68 ± 0.43^a^	39.05 ± 1.26^b^	0.01
	21	41.05 ± 0.72	41.16 ± 0.82	41.14 ± 0.88	41.23 ± 1.19	0.99
ALP (U/L)	1	49.88 ± 2.39^a^	38.50 ± 1.92^b^	42.38 ± 2.19^ab^	47.75 ± 4.55^a^	0.04
	21	37.88 ± 2.66	44.88 ± 6.26	38.63 ± 2.67	44.63 ± 3.58	0.47
AMM (μmol/L)	1	123.34 ± 4.40^a^	74.89 ± 6.69^b^	62.10 ± 6.66^b^	107.64 ± 3.35^a^	<0.01
	21	93.73 ± 1.45^a^	52.00 ± 2.06^b^	58.53 ± 3.84^b^	59.26 ± 7.20^b^	<0.01
HDL-C (mmol/L)	1	0.64 ± 0.03	0.63 ± 0.03	0.62 ± 0.03	0.71 ± 0.03	0.16
	21	0.72 ± 0.04^b^	0.91 ± 0.06^a^	0.72 ± 0.04^b^	0.95 ± 0.04^a^	<0.01
LDL-C (mmol/L)	1	0.80 ± 0.06	0.86 ± 0.07	0.81 ± 0.04	0.80 ± 0.03	0.83
	21	1.10 ± 0.13	1.19 ± 0.05	0.96 ± 0.08	1.08 ± 0.05	0.31
TC (mmol/L)	1	1.41 ± 0.07	1.40 ± 0.09	1.35 ± 0.04	1.47 ± 0.05	0.67
	21	1.82 ± 0.11	2.02 ± 0.09	1.63 ± 0.11	1.91 ± 0.07	0.06
TG (mmol/L)	1	0.28 ± 0.03	0.23 ± 0.02	0.25 ± 0.02	0.24 ± 0.04	0.74
	21	0.19 ± 0.03	0.21 ± 0.03	0.17 ± 0.01	0.15 ± 0.02	0.27
TP (g/L)	1	67.85 ± 1.00^b^	70.15 ± 0.99^ab^	70.45 ± 0.63^ab^	73.40 ± 1.65^a^	0.02
	21	75.66 ± 1.42	78.83 ± 1.61	76.79 ± 2.02	82.33 ± 1.82	0.06
UN (mmol/L)	1	4.19 ± 0.30	4.54 ± 0.16	5.35 ± 0.30	4.69 ± 0.35	0.06
	21	4.83 ± 0.19	5.88 ± 0.54	5.65 ± 0.65	5.79 ± 0.32	0.37

*Data are presented as means with SE (n = 8)*.

a, b *Mean values in the same row with different superscripts were significantly different (P < 0.05). The BS-A+B, BS-A+BL, and BS-B+BL groups contained 125 g/t Bacillus subtilis A (BS-A), 125 g/t Bacillus subtilis B (BS-B), and/or 125 g/t Bacillus licheniformis (BL), respectively*. *ALB, albumin; ALP, alkaline phosphatase; AMM, ammonia; HDL-C, high-density lipoprotein-cholesterol; LDL-C, low-density lipoprotein-cholesterol; TC, total cholesterol; TG, triglyceride; TP, total protein; UN, urea nitrogen*.

### Amount and Composition of Fecal Microbiota

The effects of different dietary probiotic mixture supplementation on fecal microbiota composition in sows are summarized in Table 7. No significant differences (*P* > 0.05) were observed in the amounts of *Bifidobacterium* spp., *E. coli*, and total bacteria in the fecal samples from the different treatment groups. The ratio of fecal *Lactobacillus* to *E. coli* on day 7 postpartum was increased (*P* < 0.05) in the BS-A+BL group, and the amount of *Lactobacillus* tended to increase (*P* = 0.07), when compared with the CON and BS-B+BL groups. The fecal amount of *Firmicutes* was decreased (*P* < 0.05) in the BS-B+BL group compared with the CON and BS-A+BL groups on day 7 postpartum and tended to increase (*P* = 0.07) in the probiotic mixture supplemented groups on day 21 postpartum. The fecal amount of *Clostridium cluster* IV in the BS-B+BL group tended to increase (*P* = 0.05) on day 112 of pregnancy compared with the CON group.

**Table 7:** Effects of dietary probiotic mixture supplementation on fecal microbiota quantity in sows.

**Items (Lg copies/g)**	**CON group**	**BS-A+B group**	**BS-A+BL group**	**BS-B+BL group**	***P*-values**
***Bifidobacterium***					
Day 100 of pregnancy	5.63 ± 0.38	5.93 ± 0.39	6.10 ± 0.30	6.03 ± 0.34	0.80
Day 112 of pregnancy	4.93 ± 0.27	5.37 ± 0.40	4.60 ± 0.48	5.00 ± 0.33	0.56
Day 7 postpartum	4.60 ± 0.41	4.57 ± 0.42	4.76 ± 0.41	4.03 ± 0.31	0.59
Day 14 postpartum	4.30 ± 0.27	4.43 ± 0.30	4.71 ± 0.25	3.97 ± 0.30	0.32
Day 21 postpartum	4.37 ± 0.44	4.47 ± 0.27	4.89 ± 0.37	4.46 ± 0.27	0.72
***Lactobacillus***					
Day 100 of pregnancy	7.12 ± 0.28	7.58 ± 0.31	7.09 ± 0.45	7.04 ± 0.48	0.75
Day 112 of pregnancy	6.28 ± 0.32	6.70 ± 0.27	5.99 ± 0.47	6.40 ± 0.31	0.56
Day 7 postpartum	5.54 ± 0.41	6.13 ± 0.41	6.75 ± 0.31	5.56 ± 0.24	0.07
Day 14 postpartum	6.90 ± 0.32	6.66 ± 0.23	6.80 ± 0.23	6.78 ± 0.33	0.95
Day 21 postpartum	6.61 ± 0.41	6.39 ± 0.33	5.59 ± 0.53	7.08 ± 0.25	0.08
***Escherichia coli***					
Day 100 of pregnancy	6.49 ± 0.36	6.27 ± 0.32	6.17 ± 0.35	6.01 ± 0.28	0.77
Day 112 of pregnancy	7.37 ± 0.18	7.17 ± 0.18	7.65 ± 0.15	7.52 ± 0.18	0.25
Day 7 postpartum	7.95 ± 0.24	7.65 ± 0.18	7.43 ± 0.14	7.70 ± 0.12	0.45
Day 14 postpartum	7.46 ± 0.24	7.33 ± 0.18	7.03 ± 0.19	7.27 ± 0.22	0.52
Day 21 postpartum	6.89 ± 0.24	6.52 ± 0.22	6.36 ± 0.28	6.90 ± 0.32	0.41
***Lactobacillus/E. coli***					
Day 100 of pregnancy	1.13 ± 0.09	1.23 ± 0.09	1.15 ± 0.06	1.20 ± 0.12	0.85
Day 112 of pregnancy	0.86 ± 0.05	0.94 ± 0.06	0.78 ± 0.06	0.86 ± 0.06	0.31
Day 7 postpartum	0.70 ± 0.06^b^	0.80 ± 0.05^ab^	0.91 ± 0.05^a^	0.73 ± 0.04^b^	0.02
Day 14 postpartum	0.94 ± 0.07	0.92 ± 0.06	0.97 ± 0.05	0.94 ± 0.06	0.92
Day 21 postpartum	0.97 ± 0.07	1.00 ± 0.07	0.91 ± 0.11	1.04 ± 0.07	0.69
***Clostridium cluster*** **IV**					
Day 100 of pregnancy	7.50 ± 0.14	7.83 ± 0.08	7.70 ± 0.10	7.75 ± 0.08	0.16
Day 112 of pregnancy	6.69 ± 0.14	6.90 ± 0.13	6.94 ± 0.17	7.24 ± 0.09	0.05
Day 7 postpartum	7.10 ± 0.19	6.93 ± 0.08	6.86 ± 0.12	6.90 ± 0.10	0.58
Day 14 postpartum	6.95 ± 0.10	7.01 ± 0.11	6.91 ± 0.19	6.72 ± 0.16	0.54
Day 21 postpartum	6.79 ± 0.16	6.85 ± 0.15	6.84 ± 0.12	7.04 ± 0.10	0.59
***Firmicutes***					
Day 100 of pregnancy	9.20 ± 0.23	9.70 ± 0.09	9.70 ± 0.10	9.25 ± 0.42	0.32
Day 112 of pregnancy	8.46 ± 0.23	8.75 ± 0.15	8.74 ± 0.06	8.60 ± 0.21	0.62
Day 7 postpartum	8.85 ± 0.16^a^	8.60 ± 0.14^ab^	8.91 ± 0.17^a^	8.26 ± 0.14^b^	0.02
Day 14 postpartum	8.98 ± 0.09	9.11 ± 0.07	8.68 ± 0.24	8.72 ± 0.09	0.12
Day 21 postpartum	7.40 ± 0.43	8.30 ± 0.20	8.36 ± 0.51	8.78 ± 0.17	0.07
***Total bacteria***					
Day 100 of pregnancy	9.09 ± 0.22	9.39 ± 0.11	8.88 ± 0.42	8.97 ± 0.41	0.69
Day 112 of pregnancy	9.01 ± 0.14	9.12 ± 0.07	9.05 ± 0.14	9.28 ± 0.07	0.35
Day 7 postpartum	9.30 ± 0.07	9.02 ± 0.08	9.10 ± 0.15	9.02 ± 0.05	0.16
Day 14 postpartum	9.29 ± 0.06	9.33 ± 0.06	9.30 ± 0.11	8.74 ± 0.32	0.10
Day 21 postpartum	9.31 ± 0.10	9.13 ± 0.09	9.34 ± 0.08	9.30 ± 0.10	0.36

*Data are presented as means with SE (n = 8)*.

a, b *Mean values in the same row with different superscripts were significantly different (P < 0.05). The BS-A+B, BS-A+BL, and BS-B+BL groups contained 125 g/t Bacillus subtilis A (BS-A), 125 g/t Bacillus subtilis B (BS-B), and/or 125 g/t Bacillus licheniformis (BL), respectively*.

### Fecal Concentrations of Bacterial Metabolites

The effects of different dietary probiotic mixture supplementation on fecal SCFA concentrations in sows are presented in Table 8. On day 100 of pregnancy, the fecal valerate concentration was higher (*P* < 0.05) in the BS-A+BL and BS-B+BL groups compared with the CON group. The fecal isobutyrate and branched-chain fatty acid (BCFA) concentrations were higher (*P* < 0.05) in the three probiotic mixture supplemented groups compared with the CON group. Moreover, the fecal isobutyrate concentration in the BS-B+BL group and the BCFA concentration in the BS-A+BL and BS-B+BL groups were higher (*P* < 0.05) compared with the BS-A+B group. However, the fecal acetate (*P* = 0.08) concentration tended to increase in the probiotic mixture supplemented groups compared with the CON group. On day 112 of pregnancy, a higher (*P* < 0.05) propionate concentration was observed in the BS-A+B group compared with the CON group. The fecal straight-chain fatty acids, isovalerate, and total SCFA concentrations in the BS-A+BL and BS-B+BL groups and the valerate and BCFA concentrations in the BS-A+BL group were lower (*P* < 0.05) when compared with the BS-A+B group. Moreover, the acetate concentration tended to increase in the BS-A+B group (*P* = 0.09) compared with the other three groups. On day 7 postpartum, the fecal acetate, isovalerate, and BCFA concentrations were higher (*P* < 0.05) in the BS-A+B and BS-A+BL groups compared with the CON group. Moreover, the straight-chain fatty acid concentration in the BS-A+B and the total SCFA concentration in the probiotic mixture supplemented groups were higher (*P* < 0.05) when compared with the CON group. However, no significant differences (*P* > 0.05) were observed in the fecal bacterial metabolites on days 14 and 21 postpartum among the different treatment groups.

**Table 8:** Effects of dietary probiotic mixture supplementation on fecal short-chain fatty acids (SCFA) concentrations in sows.

**Items (mg/g)**	**CON group**	**BS-A+B group**	**BS-A+BL group**	**BS-B+BL group**	***P*-values**
**Acetate**					
Day 100 of pregnancy	3.87 ± 0.35	4.61 ± 0.21	4.81 ± 0.50	5.15 ± 0.22	0.08
Day 112 of pregnancy	5.59 ± 0.43	6.20 ± 0.12	5.07 ± 0.32	4.87 ± 0.56	0.09
Day 7 postpartum	5.36 ± 0.36^b^	6.33 ± 0.18^a^	6.56 ± 0.29^a^	6.10 ± 0.24^ab^	0.03
Day 14 postpartum	7.16 ± 0.78	7.74 ± 0.42	7.30 ± 0.50	7.06 ± 0.50	0.84
Day 21 postpartum	5.29 ± 0.29	5.57 ± 0.26	5.30 ± 0.17	5.27 ± 0.21	0.78
**Propionate**					
Day 100 of pregnancy	1.96 ± 0.14	2.34 ± 0.13	2.18 ± 0.11	2.23 ± 0.12	0.20
Day 112 of pregnancy	2.30 ± 0.24^b^	3.00 ± 0.15^a^	2.07 ± 0.10^b^	2.31 ± 0.14^b^	<0.01
Day 7 postpartum	2.32 ± 0.21	3.12 ± 0.32	2.50 ± 0.16	2.41 ± 0.26	0.11
Day 14 postpartum	3.24 ± 0.60	3.81 ± 0.26	3.59 ± 0.42	3.10 ± 0.23	0.58
Day 21 postpartum	2.28 ± 0.13	2.56 ± 0.17	2.36 ± 0.13	2.42 ± 0.12	0.54
**Butyrate**					
Day 100 of pregnancy	0.41 ± 0.13	0.47 ± 0.20	0.07 ± 0.01	0.48 ± 0.27	0.36
Day 112 of pregnancy	0.19 ± 0.10	0.10 ± 0.01	0.11 ± 0.01	0.32 ± 0.13	0.23
Day 7 postpartum	0.34 ± 0.12	0.44 ± 0.22	0.11 ± 0.01	0.17 ± 0.06	0.29
Day 14 postpartum	1.60 ± 0.38	1.70 ± 0.10	1.75 ± 0.32	1.76 ± 0.36	0.98
Day 21 postpartum	1.25 ± 0.11	1.45 ± 0.06	1.16 ± 0.09	1.41 ± 0.09	0.10
**Valerate**					
Day 100 of pregnancy	0.22 ± 0.02^b^	0.25 ± 0.02^ab^	0.30 ± 0.04^a^	0.33 ± 0.02^a^	0.04
Day 112 of pregnancy	0.32 ± 0.04^ab^	0.38 ± 0.04^a^	0.25 ± 0.01^b^	0.29 ± 0.02^ab^	0.03
Day 7 postpartum	0.26 ± 0.02	0.41 ± 0.03	0.44 ± 0.11	0.33 ± 0.02	0.19
Day 14 postpartum	0.45 ± 0.05	0.49 ± 0.02	0.44 ± 0.07	0.42 ± 0.04	0.83
Day 21 postpartum	0.33 ± 0.02	0.37 ± 0.03	0.30 ± 0.01	0.37 ± 0.02	0.12
**Straight-chain fatty acids**					
Day 100 of pregnancy	5.60 ± 0.51	6.24 ± 0.63	6.06 ± 1.09	5.70 ± 0.28	0.91
Day 112 of pregnancy	8.40 ± 0.64^ab^	9.68 ± 0.23^a^	7.51 ± 0.40^b^	7.22 ± 0.75^b^	0.02
Day 7 postpartum	8.29 ± 0.39^b^	10.30 ± 0.46^a^	9.61 ± 0.47^ab^	9.01 ± 0.45^ab^	0.02
Day 14 postpartum	12.44 ± 1.51	13.72 ± 0.71	13.07 ± 1.27	12.34 ± 1.07	0.83
Day 21 postpartum	9.16 ± 0.48	9.94 ± 0.46	9.12 ± 0.33	9.46 ± 0.36	0.48
**Isobutyrate**					
Day 100 of pregnancy	0.73 ± 0.19^c^	1.32 ± 0.17^b^	1.69 ± 0.15^ab^	2.09 ± 0.08^a^	<0.01
Day 112 of pregnancy	0.31 ± 0.02	0.34 ± 0.01	0.25 ± 0.01	0.41 ± 0.11	0.24
Day 7 postpartum	0.27 ± 0.01	0.37 ± 0.03	0.35 ± 0.05	0.29 ± 0.02	0.06
Day 14 postpartum	0.43 ± 0.05	0.48 ± 0.03	0.40 ± 0.05	0.39 ± 0.03	0.41
Day 21 postpartum	0.32 ± 0.02	0.36 ± 0.03	0.31 ± 0.01	0.36 ± 0.02	0.23
**Isovalerate**					
Day 100 of pregnancy	0.40 ± 0.04	0.46 ± 0.03	0.57 ± 0.08	0.57 ± 0.04	0.06
Day 112 of pregnancy	0.57 ± 0.05^ab^	0.66 ± 0.02^a^	0.46 ± 0.02^b^	0.49 ± 0.04^b^	<0.01
Day 7 postpartum	0.51 ± 0.04^b^	0.75 ± 0.04^a^	0.73 ± 0.11^a^	0.58 ± 0.04^ab^	0.04
Day 14 postpartum	0.85 ± 0.10	0.93 ± 0.06	0.80 ± 0.12	0.77 ± 0.06	0.59
Day 21 postpartum	0.61 ± 0.05	0.70 ± 0.07	0.58 ± 0.02	0.71 ± 0.04	0.21
**Branched-chain fatty acids (BCFA)**					
Day 100 of pregnancy	1.13 ± 0.19^c^	1.78 ± 0.17^b^	2.26 ± 0.16^a^	2.66 ± 0.10^a^	<0.01
Day 112 of pregnancy	0.87 ± 0.07^ab^	1.00 ± 0.03^a^	0.71 ± 0.03^b^	0.90 ± 0.08^a^	0.02
Day 7 postpartum	0.78 ± 0.05^b^	1.13 ± 0.07^a^	1.09 ± 0.16^a^	0.88 ± 0.05^ab^	0.03
Day 14 postpartum	1.28 ± 0.14	1.41 ± 0.09	1.20 ± 0.17	1.16 ± 0.09	0.54
Day 21 postpartum	0.93 ± 0.07	1.06 ± 0.10	0.89 ± 0.03	1.07 ± 0.06	0.19
**Total SCFA**					
Day 100 of pregnancy	6.74 ± 0.44	8.02 ± 0.55	8.31 ± 1.07	8.36 ± 0.27	0.28
Day 112 of pregnancy	9.27 ± 0.68^ab^	10.68 ± 0.26^a^	8.22 ± 0.41^b^	8.12 ± 0.69^b^	<0.01
Day 7 postpartum	9.07 ± 0.40^c^	11.43 ± 0.51^a^	10.70 ± 0.58^ab^	9.89 ± 0.46^bc^	0.04
Day 14 postpartum	13.71 ± 1.62	15.13 ± 0.77	14.27 ± 1.42	13.50 ± 1.15	0.81
Day 21 postpartum	10.08 ± 0.54	11.01 ± 0.49	10.01 ± 0.34	10.52 ± 0.39	0.38

*Data are presented as means with SE (n = 8)*.

a–c *Mean values in the same row with different superscripts were significantly different (P < 0.05). The BS-A+B, BS-A+BL, and BS-B+BL groups contained 125 g/t Bacillus subtilis A (BS-A), 125 g/t Bacillus subtilis B (BS-B), and/or 125 g/t Bacillus licheniformis (BL), respectively*.

The effects of different dietary probiotic mixture supplementation on fecal bioamine concentrations in sows are presented in Table 9. There were no significant differences in the bioamine concentrations among the different treatment groups on day 100 of pregnancy except that the tryptamine concentration tended to increase (*P* = 0.06) in the BS-A+B and BS-A+BL groups. On day 112 of pregnancy, the spermine concentration was higher (*P* < 0.05) in the BS-B+BL group compared with the other three groups. On day 7 postpartum, the fecal 1,7-heptanediamine (*P* < 0.05) and spermidine (*P* = 0.07) concentrations were higher in the BS-A+BL group compared with the other three groups. The fecal tryptamine (*P* = 0.08) concentration in the BS-B+BL group and the spermine (*P* < 0.05) concentration in the BS-A+BL and BS-B+BL groups were higher compared with the CON and BS-A+B groups on day 14 postpartum. Moreover, the 1,7-heptanediamine concentration was higher (*P* < 0.05) in the BS-A+BL group compared with the CON and BS-B+BL groups on day 21 postpartum. However, the spermine concentration tended to increase (*P* = 0.07) in the BS-B+BL group on day 21 postpartum.

**Table 9:** Effects of dietary probiotic mixture supplementation on fecal bioamine concentrations in sows.

**Items (μg/g)**	**CON group**	**BS-A+B group**	**BS-A+BL group**	**BS-B+BL group**	***P*-values**
**Tryptamine**					
Day 100 of pregnancy	2.92 ± 0.24	3.27 ± 0.38	4.39 ± 1.27	1.88 ± 0.29	0.06
Day 112 of pregnancy	3.84 ± 0.59	5.51 ± 0.95	4.35 ± 0.51	3.26 ± 0.47	0.12
Day 7 postpartum	2.16 ± 0.40	2.33 ± 0.48	2.05 ± 0.21	2.40 ± 0.30	0.90
Day 14 postpartum	1.47 ± 0.53	1.52 ± 0.29	1.86 ± 0.39	3.17 ± 0.71	0.08
Day 21 postpartum	1.55 ± 0.45	1.69 ± 0.44	1.78 ± 0.32	1.42 ± 0.08	0.93
**1,7-Heptanediamine**					
Day 100 of pregnancy	0.44 ± 0.03	0.38 ± 0.04	0.38 ± 0.07	0.33 ± 0.06	0.42
Day 112 of pregnancy	0.48 ± 0.07	0.57 ± 0.09	0.75 ± 0.18	0.64 ± 0.13	0.48
Day 7 postpartum	0.37 ± 0.05^b^	0.38 ± 0.04^b^	0.57 ± 0.05^a^	0.34 ± 0.06^b^	<0.01
Day 14 postpartum	0.29 ± 0.05	0.29 ± 0.02	0.28 ± 0.01	0.34 ± 0.04	0.34
Day 21 postpartum	0.30 ± 0.04^b^	0.37 ± 0.02^ab^	0.47 ± 0.07^a^	0.26 ± 0.03^b^	<0.01
**Spermidine**					
Day 100 of pregnancy	10.44 ± 1.71	7.13 ± 1.69	9.60 ± 2.47	13.62 ± 2.73	0.27
Day 112 of pregnancy	12.00 ± 1.28	11.51 ± 1.30	14.61 ± 2.14	15.93 ± 1.41	0.17
Day 7 postpartum	6.08 ± 0.68	6.66 ± 0.43	8.98 ± 0.78	6.82 ± 1.11	0.07
Day 14 postpartum	10.06 ± 0.98^b^	8.60 ± 0.69^b^	16.82 ± 1.78^a^	10.56 ± 1.00^b^	<0.01
Day 21 postpartum	15.27 ± 1.84	15.97 ± 1.64	16.17 ± 2.37	17.60 ± 1.81	0.86
**Spermine**					
Day 100 of pregnancy	0.72 ± 0.11	0.30 ± 0.06	0.61 ± 0.16	0.69 ± 0.18	0.13
Day 112 of pregnancy	0.67 ± 0.10^b^	0.93 ± 0.11^b^	0.95 ± 0.19^b^	1.78 ± 0.24^a^	<0.01
Day 7 postpartum	0.62 ± 0.11	0.69 ± 0.08	0.88 ± 0.10	0.55 ± 0.08	0.11
Day 14 postpartum	0.70 ± 0.06^c^	0.54 ± 0.08^c^	1.61 ± 0.26^a^	1.17 ± 0.13^b^	<0.01
Day 21 postpartum	1.18 ± 0.16	1.50 ± 0.23	1.20 ± 0.23	1.92 ± 0.21	0.07

*Data are presented as means with SE (n = 8)*.

a–c *Mean values in the same row with different superscripts were significantly different (P <0.05). The BS-A+B, BS-A+BL, and BS-B+BL groups contained 125 g/t Bacillus subtilis A (BS-A), 125 g/t Bacillus subtilis B (BS-B), and/or 125 g/t Bacillus licheniformis (BL), respectively*.

## Discussion

Dietary probiotics supplementation can maintain or even improve indicators of gut health, leading to an overall better performance and health status in animal production. Therefore, this area of research has become more and more active in the field of animal nutrition (23). The present study showed that maternal supplementation with different probiotics mixture from late pregnancy to day 21 postpartum increased the average body weight and average daily gain of weaned piglets, and BS-A+BL supplementation increased the number of weaned piglets. Similarly, Alexopoulos et al. (24) also demonstrated that 400 g/t *B. licheniformis* and *B. subtilis* supplementation from 14 days prior to the expected farrowing to weaning periods increased the number of weaned piglets per litter and the BW of piglet at weaning. In addition, maternal intestinal microflora can affect the colonization and development of gut microbiota of offspring, which is associated with the weight gain of offspring (25). Therefore, these findings indicated that different probiotic mixture supplementation to sows during late pregnancy to day 21 postpartum are able to improve the reproductive performance of sows, and thus influence the growth performance of piglets.

The nutrient composition of sows' milk is closely related to the survival rate and the growth and development of piglets (4). Several studies have reported that *Bacillus* spp. such as *B. subtilis* and *B. licheniformis* inclusion in sow diets during late gestation and lactation are able to influence the colostrum or milk composition (24, 26). The present study shows that dietary supplementation with different probiotic mixture increased the concentrations of fat and dry matter in the colostrum, as well as the concentrations of protein and UN in the BS-A+BL group. Consumption of milk with better quality has also been reported to increase the piglets' weaning weight when sows were fed *B. subtilis* during lactation (24, 26). Therefore, these findings suggest that the improvement in the reproductive performance of sows might be related to the dietary probiotic supplementation (27), which improves the sows' milk quality and quantity and promotes fat deposition and growth of suckling piglets up to a certain extent.

Plasma biochemical parameters can partly reflect the nutritional status, tissue and organ functions, and metabolic status of animals. In addition, the plasma AMM concentration may reflect the liver function of animals (28). The present study showed that dietary supplementation with different probiotic mixture decreased plasma AMM concentration on day 21 postpartum, suggesting that the nitrogen metabolism of sows was elevated. The HDL-C is responsible for transporting TC to liver cells for oxidation, the plasma concentration of which is markedly related to lipoprotein metabolism (29). The present study showed that dietary supplementation with probiotic mixture BS-A+B and BS-B+BL increased the plasma HDL-C concentration on day 21 postpartum, indicating that these probiotic mixtures improved the lipoprotein metabolism of sows. Moreover, lactating sows need higher energy reserves and nutrients to maintain body tissues and support milk production (30). Research evidence showed that dietary probiotic supplementation can improve the intestinal environment and nutrient metabolism (31), as well as backfat thickness at birth and weaning (32, 33). The present study showed that dietary BS-A+BL supplementation increased the backfat thickness with changes recorded from days 85 to 112 of pregnancy. This suggests that in sows, the recovery of physical condition postpartum is promoted up to a certain extent by the supplementation used.

The intestinal microbiota composition plays a key role in maintaining health and regulating pathogenesis in the host (34). Studies have found that the quantity of intestinal *Firmicutes* has the potential to increase the energy intake from the diet and the body weight in humans (35). Moreover, *Clostridium clusters* IV, *Lactobacillus*, and *Bifidobacterium* can participate in nutrient metabolism and energy recycling and play important roles in the trophic, metabolic, and protective functions of the host (36). Dietary probiotic supplementation could regulate the balance and activity of gut microbes and thereby affect the metabolism and utilization of nutrients (37), the physiology and immune processes, the protection against pathogens, and the resistance to disease (38). In the present study, dietary supplementation with probiotic BS-A+BL increased the *Lactobacillus* to *E. coli* ratio, which might have a beneficial effect on the reproductive performance of sows and the intestinal health of offspring. Moreover, dietary supplementation with probiotic mixture of BS-A+BL on day 7 postpartum and BS-B+BL on day 21 postpartum trended to increase the abundance of *Lactobacillus* in sows. This is in agreement with the previous study by Kaewtapee et al. (39), which reported that *Bacillus* spp. (*B. subtilis* and *B. licheniformis*) supplementation in diets with low- and high-protein content increased the abundances of *Bifidobacterium* spp. and *Lactobacillus* spp. However, these findings are not in line with those of Bohmer et al. (40), who found that the fecal bacterial counts of sows were not affected by probiotics supplementation. This discrepancy might be explained by the differences in genetic background, breeds, and ages of the sows, as well as the dose and periods of prebiotic supplementation in the different studies.

The gut microbial metabolites influence nutrient metabolism, immunity, and health of the host through various regulatory mechanisms (41, 42). Some of anaerobic bacteria in the colon ferment the complex carbohydrates, indigestible fibers, or amino acids released from proteins, producing the SCFA, such as acetate, butyrate, and propionate (43). Among these metabolites, acetate can be metabolized by peripheral tissues (44) and provide energy for the host. Propionate is primarily used by the liver and can regulate cholesterol synthesis (45). Our results showed that dietary BS-A+B supplementation increased the fecal concentrations of propionate and valerate on day 112 of pregnancy and acetate and straight-chain fatty acids on day 7 postpartum. Moreover, the concentrations of acetate on day 7 postpartum and valerate on day 100 pregnancy were higher in the BS-A+BL group, as well as the concentration of valerate on day 100 pregnancy in the BS-B+BL group. These findings suggest that dietary probiotic mixture supplementation may modulate the SCFA production in the colon of sows. A previous study reported that obesity has been found to be associated with the increase in fecal total SCFA concentration (46). However, it is unknown if a causative link exists between these two parameters. Therefore, it has been postulated that the probiotic strains may provide the additional energy for the host to promote weight gain in sows (47). In another study, Ohigashi et al. (48) reported that the increase in SCFA production is accompanied by a decrease in the luminal pH, which resulted in the suppression of intestinal pathogens and increased nutrient absorption. Thus, these findings indicate that the intestinal microflora balance could be improved by dietary probiotic mixture supplementation.

The BCFA, including isobutyrate and isovalerate, are the products of L-leucine, L-isoleucine, and L-valine obtained from protein breakdown. The BCFA concentrations are the markers of protein catabolism in the intestinal cavity (49). The present study showed that dietary probiotics BS-A+B and BS-A+BL supplementation increased the fecal BCFA concentrations on day 7 postpartum, suggesting that there are more indigestible proteins in the small intestine which entered the colon, and that the catabolism of L-leucine, L-isoleucine, or L-valine was increased in the colon (22). However, the underlying mechanisms need to be further clarified.

Bioamines are mainly produced through the decarboxylation of different amino acid precursors (including methionine, tryptophan, arginine, and ornithine) by bacterial metabolism (50, 51). These metabolites have some known physiological functions in different tissues of the body, including regulation of gene expression, nucleic acid and protein synthesis, cell signaling, cell proliferation and differentiation, and placental growth and embryonic development in animals (52). Tryptophan is linked to tryptamine via tryptophan decarboxylase, and putrescine is synthesized indirectly from arginine or directly from ornithine, which can occur simultaneously in many bacteria (53). Polyamines synthesized by the intestinal microbiota are known to be involved in intestinal epithelium renewal (54). The present study showed that the fecal concentrations of tryptamine, 1,7-heptanediamine, spermidine, and spermine were increased in the BS-A+BL group, as well as tryptamine and spermine in the BS-B+BL group. These changes may be due to an increased metabolic capacity of the intestinal microbiota for amino acid decarboxylation. Previous studies demonstrated that higher levels of bioamines may contribute to decreased colonic chronic inflammation by inhibiting inflammatory cytokine synthesis in macrophages (55, 56). Further studies are necessary to determine whether the parameters of intestinal mucosal inflammation were modified by *B. subtilis* or *B. licheniformis* supplementation.

## Conclusion

Collectively, dietary supplementation with different probiotic mixture of *Bacillus* spp. in sows from late pregnancy to day 21 postpartum can increase the BW and average daily gain of offspring piglets, while only *B. subtilis* A in combination with *B. licheniformis* can increase the number of piglets. The colostrum composition was also found to be improved following dietary probiotic supplementation, an improvement that may be linked to the positive effect of piglet's growth and development. Furthermore, dietary supplementation with *B. subtilis* A in combination with *B. licheniformis* altered the intestinal microbiota and different bacterial metabolite concentrations. Further future studies will help to understand better the causal links between these different biological and biochemical parameters. Finally, it is worth noting that dietary supplementation with *B. subtilis* A in combination with *B. licheniformis* from day 85 of pregnancy to day 21 of postpartum was the optimum probiotic mixture beneficial for both sows and piglets.

## Data Availability Statement

The datasets presented in this study can be found in online repositories. The names of the repository/repositories and accession number(s) can be found in the article/supplementary material.

## Ethics Statement

The animal study was reviewed and approved by Animal Care and Use Committee of Institute of Subtropical Agriculture, Chinese Academy of Sciences.

## Author Contributions

LH and PH performed the experiments. LH and MA performed the statistical analyses and wrote the manuscript. WZ, WW, and XK contributed to experimental concepts and design, provided scientific direction, and finalized the manuscript with the help of FB. All authors read and approved the final manuscript.

## Funding

The present study was jointly supported by Special Funds for Construction of Innovative Provinces in Hunan Province (2019RS3022) and Collaboration Project of Evonik (China) Co., Ltd, China.

## Conflict of Interest

WZ was employed by the company Evonik Degussa (China). The remaining authors declare that the research was conducted in the absence of any commercial or financial relationships that could be construed as a potential conflict of interest.

## Publisher's Note

All claims expressed in this article are solely those of the authors and do not necessarily represent those of their affiliated organizations, or those of the publisher, the editors and the reviewers. Any product that may be evaluated in this article, or claim that may be made by its manufacturer, is not guaranteed or endorsed by the publisher.
